# Across‐the‐World Automated Optimization and Continuous‐Flow Synthesis of Pharmaceutical Agents Operating Through a Cloud‐Based Server

**DOI:** 10.1002/anie.201809080

**Published:** 2018-10-24

**Authors:** Daniel E. Fitzpatrick, Timothé Maujean, Amanda C. Evans, Steven V. Ley

**Affiliations:** ^1^ Department of Chemistry University of Cambridge Lensfield Road Cambridge UK; ^2^ Département de Chimie Ecole Normale Supérieure Paris Saclay 94235 Cachan Cedex France; ^3^ Department of Chemistry & Biochemistry California State University Fullerton 800 N. State College Blvd. Fullerton CA 92831 USA

**Keywords:** continuous synthesis, flow chemistry, self-optimization

## Abstract

The power of the Cloud has been harnessed for pharmaceutical compound production with remote servers based in Tokyo, Japan being left to autonomously find optimal synthesis conditions for three active pharmaceutical ingredients (APIs) in laboratories in Cambridge, UK. A researcher located in Los Angeles, USA controlled the entire process via an internet connection. The constituent synthetic steps for Tramadol, Lidocaine, and Bupropion were thus optimized with minimal intervention from operators within hours, yielding conditions satisfying customizable evaluation functions for all examples.

The demands of modern‐day synthesis often go beyond the task of simply assembling a particular target molecule and include additional evaluation criteria, whereby cost, efficiency, robustness, and sustainability can also be key factors.^1^ Furthermore, this leads to the need for the discovery of greater and more diverse reactivity patterns together with improved optimization tools^2^ and other enabling technologies^3^ to facilitate levels of automation. Deeper reaction understanding, data acquisition, and mining with machine‐learning algorithms are fueling opportunities in artificial intelligence (AI) and machine intuition for example.^4^

New protocols are necessary for maximizing resource‐accelerated synthesis,^5^ which is an area where flow chemistry methods and continuous processing can demonstrate distinct advantages.^6^, ^7^ Automation of repetitive and trivial reaction sequences expedites development, leading to more efficient processing paradigms, particularly when integrated with numerous unit operations involving in‐line work‐up and reaction telescoping techniques.

Unlike previous elegant studies of multi‐step flow synthesis of natural products^8^ and active pharmaceutical ingredients (APIs),^9^, ^10^ the work reported herein breaks new ground in that there is a move away from using pre‐optimized reaction combinations towards self‐optimized processing^11^ through information feedback via reaction monitoring.

Here we demonstrate the ability to work across international borders and time domains by harnessing the Cloud through servers operating in Japan to produce three active pharmaceutical ingredients (APIs). Processes are managed from Los Angeles, USA, with equipment and chemicals located in Cambridge, UK. The autonomous nature of this set up helps with the efficient use of equipment located at remote sites to avoid redundancy. The system^12^ can be rapidly reconfigured to accommodate new reaction combinations and safe shutdown sequences, and is securely protected through appropriate firewalls and other IT security apparatus (see the Supporting Information). Compound delivery at distant sites in this fashion has consequences for future access to medicines across the world^13^ with wider applications also being possible through enhanced collaboration, including expanded access to specialized knowledge and equipment.

Further, with continued modern developments in inexpensive microcontrollers^14^, ^15^, ^16^, ^17^ and computers, such as the Raspberry Pi, a standardization of reaction protocols can be established through the use of an automated control system. Such a process would integrate with the future Internet of Chemical Things,^18^ potentially improving reproducibility and data collection for experimentalists, feeding deep‐learning algorithms of the future.

To demonstrate the utility of the approach, tramadol (**3**), lidocaine (**8**), and bupropion (**12**) were studied as representative agents.

We began our study with the preparation of (±)‐tramadol (**3**). The most common synthetic pathway to this compound follows two steps: the formation of amine **1** via a Mannich condensation, followed by a Grignard addition to yield the final product **3** as a mixture of diastereomers. For this first example, we focused solely on the second addition step, as this has been the subject of continued investigation.^19^, ^20^

A straightforward equipment layout was constructed (Figure 1 a), consisting of two reagent supply lines, a 20 mL reactor coil, and a FlowIR unit to provide spectroscopic performance feedback. Additional valves and solvent reservoirs were included with the supply lines to facilitate a reactor flushing step to minimize risk of disruption arising from solid aggregation (see the Supporting Information for more information).

**Figure 1:**
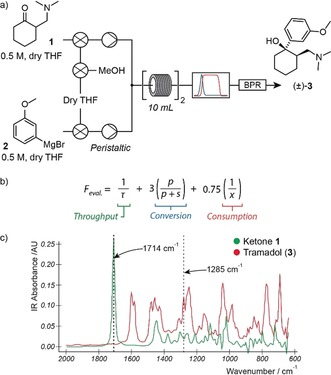
a) Equipment layout for the three‐dimensional self‐optimization of tramadol (**3**). Feedback from an inline infrared spectrometer (IR) was used by the control system to drive optimization. b) The evaluation function for the optimization of tramadol (**3**), where *τ* is residence time, *p* is product IR absorbance (compound **3**), *s* is starting material IR absorbance (compound **1**), and *x* is the equivalents of Grignard reagent **2** supplied to the reactor. c) Overlaid IR spectra of target product **3** and starting material **1**, and wavenumbers monitored for each.

The Los Angeles based operator configured the system settings to optimize for three parameters: temperature (between 30–70 °C); residence time (5–20 min); and equivalents of Grignard **2** to ketone **1** (0.5–1.6 equiv). For this reaction the control system did not just optimize for conversion, but also for material throughput and starting material consumption (Figure 1 b). The primary feedback parameter, namely conversion, was established by comparing the ratio of the IR absorption peaks corresponding to ketone **1** and target product **3** (Figure 1 c). The complex method^21^ was used to drive the optimization process for this reaction, and in all subsequent examples.

The system autonomously conducted nine experimental trials over the space of three hours (see the Supporting Information), which identified conditions that gave 86 % yield (NMR) during one hour of steady‐state operation. In this case, operating at 41 °C with a residence time of 10.9 min and with 1.6 equivalents of Grignard reagent gave the most favorable result, equivalent to a production rate of 1651 drug doses per day.

The volumetric yield^22^ for this process of 0.172 g mL^−1^ h^−1^ represented a significant improvement over a comparable flow process optimized previously by Rencurosi et al. (0.045 g mL^−1^ h^−1^).^20^

In the next example lidocaine (**8**), a local anesthetic, was produced under full self‐optimization conditions via a two‐step synthesis process (Figure 2 a). Producing this compound in flow provides a number of benefits, including improved handling of particularly hazardous reagents such as acid chloride **5** and increased thermal control.

**Figure 2:**
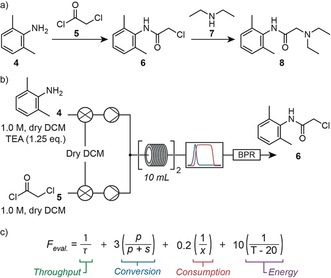
a) The modern synthesis route to lidocaine (**8**) follows a two‐step process. b) Equipment layout used for the self‐optimization of the first step to lidocaine (**8**). c) Four‐term evaluation function used to optimize the first step, where *τ* is residence time, *p* is product IR absorbance (chloroacetamide **6**), *s* is starting material IR absorbance (amine **4**), *x* is equivalents of acid chloride **5** supplied, and *T* is reactor temperature.

Overall yields obtained when conducting this synthesis under flow conditions vary greatly. Raston et al. reported an overall yield of 85 % for the segmented telescoping^23^ of both steps, where no intermediate purification was performed between reaction steps but differences in reaction flow rates prevented continuous telescoping.^24^ This yield reduced to 15 % when modified conditions for a fully telescoped process were implemented. More recently, Jamison et al. reported a 90 % yield of isolated product of lidocaine (**8**) for a fully telescoped process.^9^ Each of the fully continuous processes could produce lidocaine (**8**) at rates averaging 22.5 g day^−1^ and 16.2 g day^−1^, respectively. We set out to test whether an automated self‐optimizing approach would produce improved results notably with minimal researcher intervention.

We began these efforts with the acylation of 2,6‐dimethylaniline (**4**) with chloroacetyl chloride (**5**) to give intermediate **6**. Equipment layout resembled that used previously for tramadol (Figure 2 b). As in this first example, three parameters were selected for optimization: temperature (40–130 °C), residence time (5–25 min), and equivalents of acid chloride **5** to amine **4** (0.8–2.5 equiv). Apart from the throughput, conversion, and consumption terms in the evaluation function, we also included an energy term to encourage the unsupervised system to consider the energy impact of the reaction process (Figure 2 c).

Within 2 hours 40 minutes, the system identified conditions (105 °C, 5.0 min, 1.98 equiv of **5**; see the Supporting Information) that gave an 87 % yield of isolated product. The system was then left to run at steady state for 2.5 h to produce 39.7 g (201 mmol) of intermediate **6**, a portion of which was used for the next optimization. Thus, within one working day, the control system had moved from unoptimized conditions to a set up that produced almost 40 g of material, with minimal intervention from researchers.

The second step consisted of an amine alkylation, where intermediate **6** was reacted with diethylamine (**7**) to give lidocaine (**8**). The equipment layout was similar to the first stage (Figure 3 a), with differences arising only in the composition of feedstock solutions and reservoirs. We wished to test how our version of complex implementation would react to a scenario where chemists specifically chose to maximize the production rate of the target compound. Thus, the evaluation function contained throughput and conversion terms (Figure 3 b). As for the previous examples, temperature (70–130 °C), residence time (5–30 min), and stoichiometry (1.0–4.0 equiv of amine **7** to chloroacetamide **6**) were optimized.

**Figure 3:**
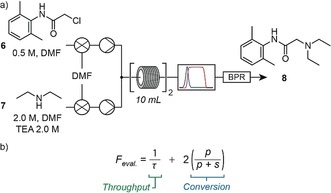
a) Equipment layout for the optimization of the amine alkylation reaction to form lidocaine (**8**). b) The evaluation function for the amine alkylation reaction to lidocaine (**8**), consisted of throughput and conversion terms only (*τ* is residence time, *p* is product IR absorbance, *s* is starting material **6** IR absorbance).

This optimization process spanned three hours, during which the control system conducted ten iterations (see the Supporting Information), identifying an optimum at a reactor temperature of 99 °C, residence time of 17.8 min, and 3.9 equiv of amine **7** to chloroacetamide **6**. This optimum gave 98 % yield of isolated product. A large amount of material remained in the feed reservoirs and so the system was left operating under these optimized conditions for four hours, allowing 15.7 g of lidocaine (**8**) to be isolated following purification.

We were therefore very pleased that the system had identified optimal conditions for the two‐step synthesis (85 % overall yield) within two working days.

In the final example, we wished to demonstrate how effectively and rapidly the system would perform when moving from unexplored conditions to a fully telescoped optimized process for a two‐step synthesis. Bupropion (**12**), a smoking cessation aid and anti‐depressant, presented an excellent opportunity to showcase the platform's capabilities.

The first step of the synthetic route to bupropion (**12**) consisted of the α‐bromination of 3′‐chloropropiophenone (**9**) to yield the intermediate bromide **10**. Given the corrosive nature of bromine solution when in contact with stainless steel equipment, an inert peristaltic pump replaced an HPLC pump used for previous optimizations (Figure 4 a). The system incorporated IR feedback in the evaluation function (Figure 4 b), monitoring the shift in characteristic peaks between the ketone **9** (1216 cm^−1^) and brominated product **10** (1300 cm^−1^). Here, the system manipulated temperature (30–80 °C), residence time (5–20 min), and stoichiometry (0.95–2.0 equiv of bromine to starting material **9**) to drive optimization. Within three hours, the system performed nine experiments identifying conditions which gave 95 % yield (44 °C, 9.7 min, and 0.95 equiv bromine), corresponding to 8.1 g h^−1^ of the target material.

**Figure 4:**
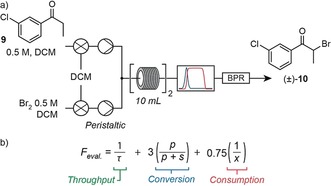
a) Equipment layout for the three‐dimensional self‐optimization of the first step to bupropion. b) The evaluation function consisted of three terms, where *τ* is residence time, *p* is product IR absorbance (bromide **10**), *s* is starting material IR absorbance (ketone **9**), and *x* is equivalents of bromine supplied to the reactor.

With these results in hand, the system was then reconfigured to optimize the second and final step which consisted of the amine alkylation of intermediate **10** with *tert*‐butylamine. Initial attempts to perform this reaction in DCM yielded poor results, which were improved through the use of *N*‐methyl‐2‐pyrrolidone (NMP; Figure 5 a).

**Figure 5:**
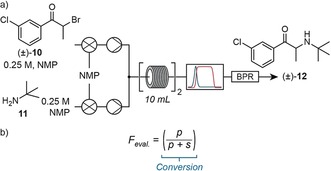
a) Equipment layout for the optimization of the amine alkylation reaction to form bupropion (**12**). b) Single‐term evaluation function for the final step to bupropion (**12**), where *p* is product IR absorbance and *s* is starting material IR absorbance (bromide **10**).

As for the first step, temperature (30–90 °C), residence time (5–30 min), and stoichiometry (0.95–3.0 equiv of *tert*‐butylamine (**11**) to bromide **10**) were chosen as parameters to drive optimization. For this case, we wished to observe how the system would react when only conversion was optimized (Figure 5 b). Within 4.5 h, the system optimized to 80 % yield (90 °C, 20 min, 3.0 equiv) having performed 11 experiments. These conditions allowed for the production of 0.72 g h^−1^ of bupropion.

The final challenge was to telescope both synthetic steps together, enabling the continuous production of bupropion. Although conditions for the first and last segments had been identified through the self‐optimization processes, suitable workup actions were still required to process the crude reaction mixture from the first step into a form that was compatible with the second. To achieve this, crude bromination reaction mixture was mixed vigorously with an aqueous sodium bisulfite stream. The organic phase, containing the bromide intermediate, was then mixed with an NMP stream and directed into a thin‐film evaporation column (see the Supporting Information), where 87 % of DCM was removed (molar basis). The resulting NMP‐enriched stream was passed to the second reactive step.

The telescoped process consisted of four‐unit operations which could be separated into three segments (Figure 6 a): α‐bromination, inter‐stage workup, and the final amine alkylation transformation.

**Figure 6:**
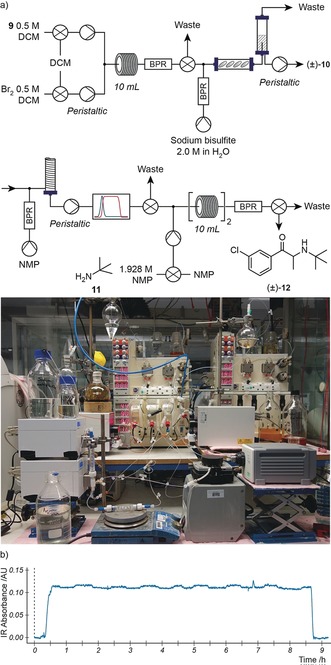
a) The telescoped synthesis of bupropion (**12**) consisted of two reaction steps and two downstream processing steps, and involving 7 items of equipment. b) Data collected from the FlowIR during steady‐state operation process, which includes the effects of a process disturbance at about 6.9 h.

Two modifications were made to the reaction conditions to maximize efficiency of the telescoped process. First, the ratio of bromine to ketone **9** in the first step was raised to 1.0 to push the reaction towards quantitative yield. Second, the concentration of amine **11** in the feed reservoir was set to 1.928 m to align optimized reaction conditions with the concentration of bromide **10** leaving the evaporation unit.

The control system was configured to follow three distinct stages, corresponding to process start‐up, steady‐state operation, and process shutdown (see the Supporting Information). During start‐up, IR feedback was used to detect the presence of the bromide intermediate in the workup stream and thus to trigger the start of the second reaction step. This process enabled staggering of the operations, minimizing material wastage and supervisory oversight required from operators.

Figure 6 b shows the data collected from the infrared spectrometer during all phases of the telescoped process. After an initial period of transient behavior, the first reaction and workup sequences reached steady state after approximately 0.6 h. It was interesting to observe how process disturbances propagated through the system during 8 h of steady‐state operation, giving an indication of the sensitivity that inline detectors can bring when monitoring multistage sequences.

For example, at approximately 6.9 h the NMP reservoir emptied. Although it was refilled within 3 min, during this time the feed into the evaporation unit consisted only of intermediate **10** in DCM. Thus, a disproportionate amount of this compound was added to the NMP solution that had yet to be removed from the column. This led to the 8 minute concentration fluctuation shown by the rise and fall in infrared absorbance as the system returned to steady state.

We were pleased with the results obtained for this target compound: the control system had facilitated the rapid transition from an unexplored route to an optimized and telescoped process, capable of producing bupropion at an average rate of 2.88 g h^−1^, within four working days.

In summary, we have demonstrated that the use of an automated control system, unencumbered by location or time domain, has the capability to greatly assist with drug development and synthesis, liberating researchers to spend time on more productive pursuits and assisting with ideas linked to delocalized manufacturing. This proof‐of‐concept approach was applied to previously reported synthetic routes of three API targets, in which a colleague in the US remotely initiated, monitored, and controlled self‐optimization reactions conducted using equipment in the UK, via servers in Japan. In all cases, optimized conditions were found within hours and, in the case of bupropion, it was possible to devise a fully optimized and telescoped system within four working days from the initial idea and synthesis plan.

Harnessing the Cloud for such reactions presents exciting opportunities to accelerate synthetic optimization, share standardized reaction procedures across the world, contribute to machine learning algorithms of the future, and facilitate distributed use of equipment. We have demonstrated that synthesis does not need to be trapped in typical confined environments but can be opened up to promote research collaborations, maximize resources, and establish reliable robust synthesis protocols beyond today's practices using conventional methods.

## Conflict of interest

The authors declare no conflict of interest.

## Supporting information

As a service to our authors and readers, this journal provides supporting information supplied by the authors. Such materials are peer reviewed and may be re‐organized for online delivery, but are not copy‐edited or typeset. Technical support issues arising from supporting information (other than missing files) should be addressed to the authors.

Supplementary
