# Supplementary material for: Across‐the‐World Automated Optimization and Continuous‐Flow Synthesis of Pharmaceutical Agents Operating Through a Cloud‐Based Server
Source: Angew Chem Int Ed Engl. 2018 Oct 24;57(46):15128–32. doi: 10.1002/anie.201809080 (PMC6391944; doi:10.1002/anie.201809080)
Supplement: Supplementary file 1 — Supplementary [file ANIE-57-15128-s001.pdf]

Angewandte Chemie
Eine Zeitschrift der Gesellschaft Deutscher Chemiker

Supporting Information

Across-the-World Automated Optimization and Continuous-Flow Synthesis of Pharmaceutical Agents Operating Through a Cloud-Based Server

*Daniel E. Fitzpatrick, Timothé Maujean, Amanda C. Evans, and Steven V. Ley**

anie_201809080_sm_miscellaneous_information.pdf

Supporting Information

1.0 Optimization results

1.1 Tramadol

Reactor flushing between experiments

This experiment involved the handling of a reagent sensitive to water and oxygen that can form solids when quenched. Any solids present during flow processes can lead to system instability, potentially blocking reactor coils or standard BPRs. Consequently, the control system was configured to flush the reactor system with 4.0 mL MeOH followed by 4.0 mL anhydrous THF, as supplied by valves shown in Figure 1a of the main text, between iterations. Any accumulated solids in the BPR would dissolve in the MeOH plug, while the plug of THF partially primed the system before the next iteration, preventing contact between reagents and MeOH.

Optimization performance

Data for each experiment trial are shown below. The value shown in the *Eval. Function* column corresponds to the value calculated by the control system using the evaluation function for this experiment (Figure 1b in the main text). *Iteration type* refers to the method used by the control system to select experiment conditions. For more information about this process, refer to references 12 and 21 in the main text.

Iteration	Temp. /°C	Time /min	Eq. Grignard	Iteration type	Eval. Function
**1**	55	12.5	1.05	Initial	3.5205
**2**	43	8.8	0.78	Initial	3.3223
**3**	43	16.3	0.78	Initial	3.4207
**4**	43	8.8	1.33	Initial	3.6480
**5**	51	16.3	1.33	Reflection	3.6224
**6**	56	8.8	1.60	Reflection	3.6973
**7**	60	6.9	1.60	Extension	3.7696
**8**	45	10.0	1.60	Reflection	3.7864
**9**	41	10.9	1.60	Extension	3.8889

1.2 Lidocaine

1.2.1 Step 1 - Optimization performance

Data for each experiment trial are shown below. The value shown in the *Eval. Function* column corresponds to the value calculated by the control system using the evaluation function for this experiment (Figure 2c in the main text).

Iteration	Temp. /°C	Time /min	Eq. acid chloride	Iteration type	Eval. Function
**1**	85.0	15	1.40	Initial	3.1516
**2**	62.5	10	1.10	Initial	2.8715
**3**	62.5	20	1.10	Initial	2.8005
**4**	62.5	10	1.70	Initial	3.1788
**5**	77.5	5	1.70	Reflection	3.2709
**6**	79.4	5	1.78	Extension	3.2863
**7**	87.5	10	2.00	Reflection	3.1963
**8**	100.4	5	1.95	Reflection	3.2889
**9**	105.2	5	1.98	Extension	3.2943

1.2.2 Step 2 - Optimization performance

Data for each experiment trial are shown below. The value shown in the *Eval. Function* column corresponds to the value calculated by the control system using the evaluation function for this experiment (Figure 3b in the main text).

Iteration	Temp. /°C	Time /min	Eq. acid chloride	Iteration type	Eval. Function
**1**	100	17.5	2.50	Initial	1.5764
**2**	85	11.3	1.75	Initial	1.0710
**3**	85	23.8	1.75	Initial	1.3352
**4**	85	11.3	3.25	Initial	1.3966
**5**	95	23.8	3.25	Reflection	1.7503
**6**	96	25.3	3.44	Extension	1.8212
**7**	102	11.3	4.00	Reflection	1.7853
**8**	95	22.7	4.00	Reflection	1.7829
**9**	100	15.8	4.00	Reflection	1.7528
**10**	99	17.8	3.91	Retraction	1.8217

1.3 Bupropion

1.3.1 Step 1 - Optimization performance

Data for each experiment trial are shown below. The value shown in the *Eval. Function* column corresponds to the value calculated by the control system using the evaluation function for this experiment (Figure 4b in the main text).

Iteration	Temp. /°C	Time /min	Eq. bromine	Iteration type	Eval. Function
**1**	55	12.5	1.55	Initial	3.6178
**2**	43	8.8	1.25	Initial	3.6656
**3**	43	16.3	1.25	Initial	3.5888
**4**	43	8.8	1.85	Initial	3.287
**5**	51	16.3	0.95	Reflection	3.9274
**6**	52	17.2	0.95	Extension	3.9131
**7**	56	8.8	1.25	Reflection	3.6583
**8**	45	10.0	0.95	Reflection	3.9948
**9**	44	9.7	0.95	Extension	3.9951

1.3.2 Step 2 - Optimization performance

Data for each experiment trial are shown below. The value shown in the *Eval. Function* column corresponds to the value calculated by the control system using the evaluation function for this experiment (Figure 5b in the main text).

Iteration	Temp. /°C	Time /min	Eq. amine	Iteration type	Eval. Function
**1**	65	12.5	1.98	Initial	0.5124
**2**	45	8.8	1.46	Initial	0.3704
**3**	45	16.3	1.46	Initial	0.4224
**4**	80	8.8	2.49	Initial	0.5540
**5**	82	16.3	2.49	Reflection	0.6372
**6**	90	18.1	2.74	Extension	0.6650
**7**	90	8.8	3.00	Reflection	0.6081
**8**	90	20.0	3.00	Reflection	0.6793
**9**	90	20.0	3.00	Extension	0.6774
**10**	84	20.0	2.49	Reflection	0.6658
**11**	90	20.0	3.00	Reflection	0.6786

2.0 Telescoped synthesis of bupropion

2.1 Solvent switching

More information about the thin-film evaporation column used for solvent switching purposes in the telescoped process can be found at reference SI-1.

In order to switch from DCM, used in the first step of the synthesis of bupropion, to NMP, used in the second, a range of operational parameters were trialled to identify suitable operating conditions. These are summarized in the table below. Optimal conditions correspond to entry 7 which allowed for the removal of 87 % of DCM (molar basis) from the combined solvent stream.

Entry	Temp /°C	F_{DCM} /mL min^{-1}	F_{NMP} /mL min^{-1}	Inlet $x^{a,b}$	Outlet $x^{a,b}$	% Removal DCM
**1**	70	0.5	0.5	0.602	0.317	47.2
**2**	70	1.0	0.5	0.751	0.544	27.5
**3**	90	1.0	0.5	0.751	0.262	65.1
**4**	90	1.0	1.0	0.602	0.267	55.6
**5**	100	1.0	0.5	0.751	0.099	86.8
**6**	100	1.0	1.0	0.602	0.153	74.6
**7**	110	1.0	0.5	0.751	0.099	86.8
**8**	110	1.0	1.0	0.602	0.145	75.8

[a] Molar fraction, calculated assuming 25 °C reservoir temperature; [b] Values were determined by ^{1}H NMR spectroscopy (600 MHz, $CDCl_3$), and are based on the relative integration of the peaks at δ 5.30 ppm (DCM) and 3.34 ppm (NMP).

2.2 Control strategy

There are three distinct phases to any multi-step process which must be considered when telescoping reaction sequences: process start up; continuous operation at steady state; and system shutdown. Each stage normally requires significant manual input from operators, for example to set equipment parameters following temporal or detector triggers during process start up and shutdown.

The structure of our control script was broken into three components (Figure S1), to reflect each of the control phases. An expanded equipment view is shown in Figure S2.

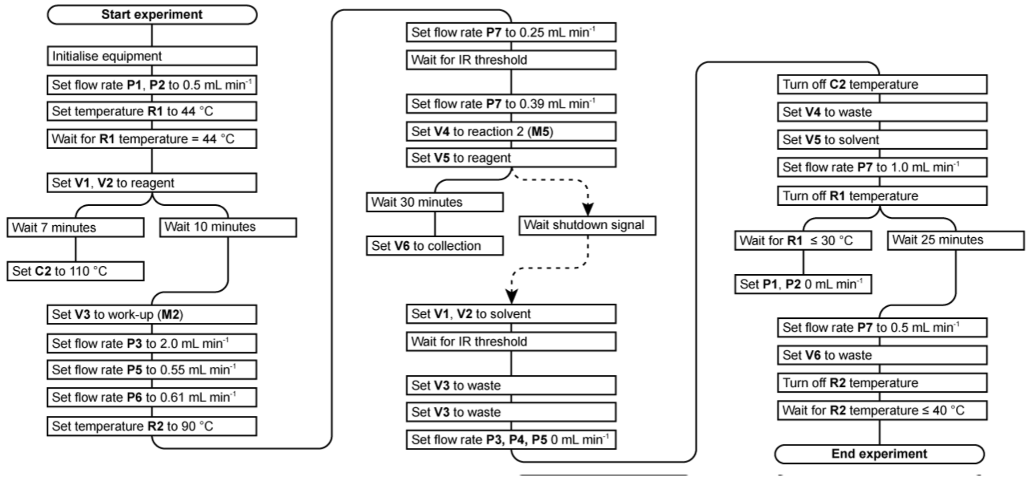

Figure S1. The automation strategy for the telescoped process was divided into three segments: the first handled process start up (items prior to dotted connection line); the second monitored conditions at steady state; and the third managed system shutdown (items after dotted connection line). System shutdown was initiated by the operator using the control system interface.

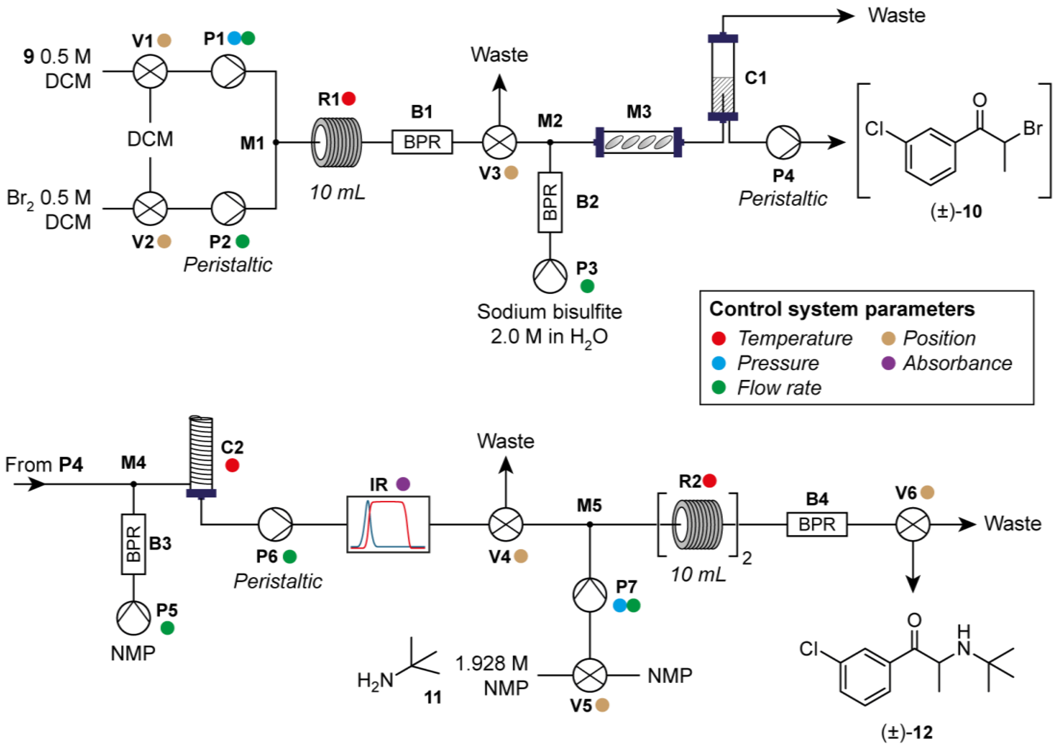

Figure S2. Schematic of the telescoped synthesis of bupropion (12).

2.2.1 *Start up*

Our scripts first began by defining experiment variables and initializing equipment connected to our control system. The temperature of R1 was set and P1 and P2 were configured to pump solvent while coils were heated to maintain back pressure. When reactor temperature had stabilized, V1 and V2 were switched to reagent lines.

The initiation of our workup sequence began seven minutes later with the heating of D1. After three minutes the script turned on P3, P5 and P6, changed the position of V3 to direct material leaving R1 into the workup segment, and began the process of warming R2 coils for the amine alkylation reaction. P7 was set to pump solvent through R2 during this time, again to maintain back pressure. Simultaneously a new listener was created to monitor IR for the presence of intermediate 10 in the solution stream leaving C2.

This listener was triggered when the absorbance of the target wavelength rose above a 0.07 AU threshold for 60 seconds. At this time the position of V4 was switched to direct solution to M5, the solvent feed of P7 was replaced with tert-butylamine (11) solution and a listener created to monitor the value of the experiment variable *startshutdownsequence*. After a period of 30 minutes, the control system directed V6 to begin collecting bupropion (12); our system had now reached steady state.

2.2.2 Shutdown

Process shutdown was initiated when the value of the experiment variable *startshutdownsequence* changed from 0 to 1 by the Los Angeles-based operator. When this occurred, V1 and V2 were switched to solvent positions and the system waited for the signal from IR to fall below a 0.05 AU threshold for 60 consecutive seconds.

When compound 10 was no longer present in the stream leaving C2, our control system halted the workup sequence by switching V3 to waste, turning off P3, P5 and P6, and cooling C2. The system also began cooling R1 and directed its effluent to waste. As soon as the temperature of R1 fell below 30 °C, P1 and P2 were turned off.

At this time the system also initiated the shutdown of the second reaction, by switching the feed to P7 to solvent and raising its flow rate to 1.0 mL min^{-1} to maintain the 20 minute residence time for reaction mixture still held in the coil. After a 25 minute period, material leaving B4 was sent to waste, the flow rate of P7 was lowered to 0.5 mL min^{-1} and R2 was cooled. As soon as the temperatures of both R2 reactor coils had fallen below 40 °C, all remaining equipment was turned off and the experiment terminated.

3.0 Control system security considerations

A number of measures were taken to protect the system given its international arrangement, including the creation of specific firewall rules to allow connections only from authorized machines, the encryption of data in transit between the server, equipment and users, and the encryption of all data stored at rest on the server.

Precautions were not focused solely on security of our system, however. Any internet-facing computer can provide an attack vector for malicious actors to compromise other devices connected to the same network, such as departmental servers storing research data. Thus a virtual local area network (VLAN) was created for this project, into which was placed all equipment which restricted their communication to other VLAN devices only. If a breach of our network were to occur, this set up provided sufficient isolation that no other department devices could be affected.

In order to facilitate communication between the VLAN and our remote-control system, a server (LeyVM) was set up that was connected to both the VLAN and the Internet. LeyVM was unable to communicate with other devices on the University network and was shielded from inbound connections, ensuring that it could not act as a single point of failure in our security configuration.

Before an experiment was commenced on the control system, LeyVM initiated a reverse Secure Socket Shell (SSH) tunnel to the remote server, acting effectively as a bridge connecting the server in Tokyo to the VLAN and the equipment within. While this SSH tunnel was operational our control system could send commands to VLAN-connected reaction equipment as required. The SSH tunnel was closed when experiments were not active, isolating our Cambridge VLAN from exposure to external devices. The security arrangements for our set up are summarized in Figure S3.

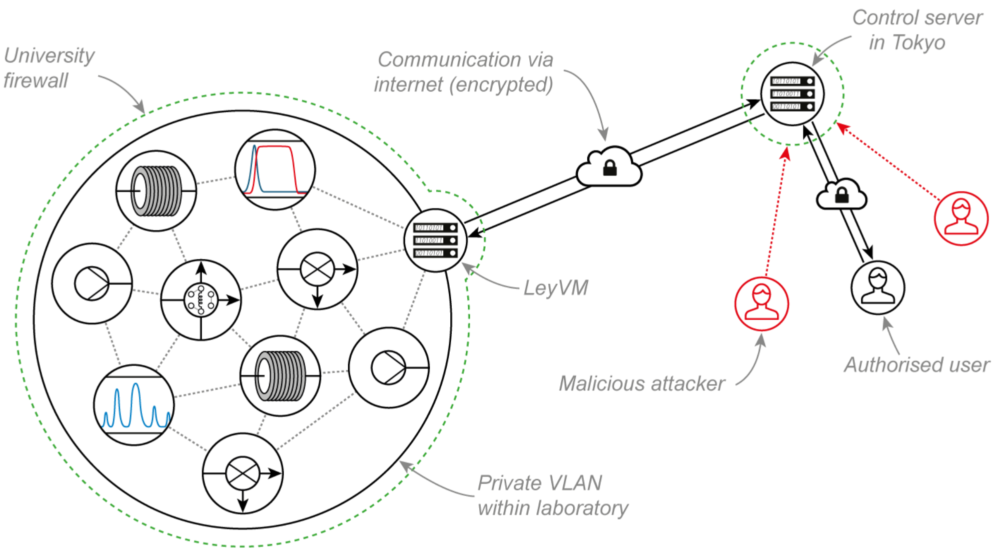

Figure S3. Schematic showing the network arrangement used for all experiments described in this work. Segregation of equipment onto an internal VLAN within the University of Cambridge network protected other department devices and shielded our equipment from outside interference. A reverse SSH tunnel encrypted communication between the Tokyo-based control server and the VLAN, while HTTPS and strong user authentication was used to protect communication between the server and authorized users.

4.0 Compound characterization

^{1}H-NMR spectra were recorded on a Bruker Avance DPX-600 spectrometer with the residual solvent peak as the internal reference ($CDCl_3$ = 7.26 ppm, d_6-DMSO = 2.50 ppm). ^{1}H resonances are reported to the nearest 0.01 ppm. ^{13}C-NMR spectra were recorded on the same spectrometers with the central resonance of the solvent peak as the internal reference ($CDCl_3$ = 77.16 ppm, d_6- DMSO = 39.52 ppm). All ^{13}C resonances are reported to the nearest 0.1 ppm. The multiplicity of 1H signals are indicated as: s = singlet, d = doublet, dd = doublet of doublet, ddd = doublet of doublet of doublet, t = triplet, q = quadruplet, sext = sextet, m = multiplet, br. = broad, or combinations of thereof. Coupling constants (J) are quoted in Hz and reported to the nearest 0.1 Hz. Where appropriate, averages of the signals from peaks displaying multiplicity were used to calculate the value of the coupling constant. Unless stated otherwise, reagents were obtained from commercial sources and used without purification. The removal of solvent under reduced pressure was carried out on a standard rotary evaporator.

Infrared spectra were recorded neat or as a thin film on a PerkinElmer Spectrum One FTIR spectrometer using Universal ATR sampling accessories. Letters in parentheses refer to the relative absorbency of the peak: w = weak, less than 30 % of the most intense peak; m = medium, ca. 31 - 69 % of the most intense peak; s = strong, greater than 70 % of the most intense peak.

Flash chromatography over silica gel was performed on a Biotage SP1 purification system with prepacked SiliCycle silica cartridges (4, 12, 25 or 40 g). Melting points were performed on a Stanford Research Systems MPA100 (OptiMelt) automated melting point system using a gradient of 1 °C min^{-1}, and are uncorrected.

High resolution mass spectrometry (HRMS) within ±5 ppm was carried out on a Waters Micromass LCT Premier spectrometer using time of flight with positive ESI, or by Mr Paul Skelton on a Bruker BioApex 47e FTICR spectrometer using positive ESI or EI at 70 eV, to within ±5 ppm of the theoretically calculated value.

LC-MS analysis was performed on an Agilent HP 1100 series chromatograph (Mercury Luma 3µ C18 column) attached to a Waters ZQ2000 mass spectrometer with ESCi ionisation source in ESI mode. Elution was carried out at a flow rate of 0.6 mL min^{-1} using a reverse phase gradient of acetonitrile and water containing 0.1 % formic acid. The gradient run is as described in the table below. Retention time (t_R) is in minutes and the *m/z* value is reported.

Time /min	% MeCN
**0.0**	5
**1.0**	5
**4.0**	95
**5.0**	95
**7.0**	5
**8.0**	5

Optical rotation measurements were recorded on a Perkin-ElmerModel 343 digital polarimeter using a Na/halogen lamp (589 nm) as the light source over a pathlength of 100 mm. $[\alpha]_D^{28.0}$ values are reported in (deg mL)(g dm)$^{-1}$ at specified concentrations (c) in g (100 mL)$^{-1}$ at temperature (T).

Elemental composition microanalysis was performed by the Microanalysis Laboratories at the Department of Chemistry, University of Cambridge and results are reported to two decimal places.

(1S,2S)-2-((dimethylamino)methyl)-1-(3-methoxyphenyl)cyclohexan-1-ol (3)

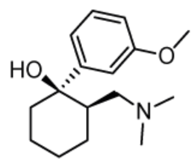

The equipment layout for this flow procedure is shown in the main text (Figure 1a).

A solution of ketone 1 (0.776 g, 5.0 mmol) in anhydrous THF (0.5 M, 10 mL) was pumped at 0.703 mL min^{-1} to a tee junction (PTFE) where it mixed with a stream of (3-methoxyphenyl)-magnesium bromide solution (1.691 g, 8.0 mmol, 1.6 eq.) in THF (0.5 M, 16 mL) pumped at 1.125 mL min^{-1}. The combined solution was directed through two 10 mL reactor coils heated to 41 °C. System pressure was maintained using a 40 psi back pressure regulator.

Crude reaction mixture was partitioned between a mixture of ice and saturated aqueous ammonium chloride solution (25 mL), and diethyl ether (25 mL). The layers were separated following vigorous mixing. Following extraction of the aqueous layer with diethyl ether (3 x 25 mL), the combined organic phase was dried over magnesium sulfate before being concentrated under reduced pressure. Residues were dissolved in toluene (25 mL) and mixed with distilled water (25 mL). An aqueous solution of hydrochloric acid (1.0 m) was added slowly until the pH of the aqueous layer lowered to 3 (by indicator paper). Following phase separation, the aqueous layer was washed with toluene (3 x 20 mL), and the organic layers discarded. The pH of the aqueous solution was adjusted to 9 (by indicator paper) by slow addition of aqueous sodium hydroxide solution (1.0 m) before ethyl acetate was added (20 mL). Following vigorous mixing, the organic layer was dried over magnesium sulfate and concentrated under reduced pressure to afford the title compound (1.133 g, 4.3 mmol, 86 % by ^{1}H NMR with internal standard, 4:1 *syn*/*anti* ratio) as a brown oil.

LC-MS t_R = 3.06 min, *m/z* 264.3;

IR (neat) v/cm^{-1}: 2935 (m), 2857 (m), 2830 (m), 2783 (w), 1599 (m), 1583 (m), 1483 (m), 1461 (s), 1430 (m), 1286 (m), 1250 (s), 1164 (m), 1092 (m), 1044 (s), 991 (s), 961 (m), 861 (m), 832 (m), 779 (s), 700 (s);

^{1}H NMR (600 MHz, $CDCl_3$) δ/ppm = 7.20 - 7.23 (m, 1H), 7.14 (bs, 1H), 7.02 (bs, 1H), 6.71 - 6.73 (m, 1H), 3.78 (s, 3H), 2.36 - 2.39 (m, 1H), 2.00 - 2.10 (m, 9H), 1.56 - 1.85 (m, 6H), 1.46 - 1.49 (m, 1H), 1.30 - 1.37 (m, 1H);

^{13}C NMR (150 MHz, $CDCl_3$) δ/ppm = 159.49, 152.01, 128.90, 117.35, 111.21, 110.94, 76.04, 61.58, 55.15, 47.77, 44.76, 41.30, 27.92, 26.86, 22.27;

HRMS *m/z* calc. for $C_{16}H_{26}NO_2$ $[M + H]^+$ 264.1964, found 264.1952, Δ = -4.0 ppm;

Microanalysis calc. (found) for $C_{16}H_{26}NO_2$ C 72.97% (70.84%), N 5.32% (5.07%), H 9.57% (9.30%).

2-Chloro-N-(2,6-dimethylphenyl)acetamide (6)

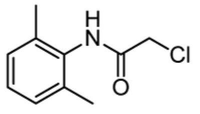

The equipment layout for this flow procedure is shown in the main text (Figure 2b).

Triethylamine (TEA) (1.74 mL, 12.5 mmol, 1.25 eq.) and 2,6-dimethylaniline (1.23 mL, 10 mmol) were added to anhydrous DCM (1.0 M 2,6-dimentylaniline, 7.03 mL). This solution was pumped at 1.342 mL min^{-1} to a tee junction where it mixed with a solution of chloroacetyl chloride (1.58 mL, 19.8 mmol, 1.98 eq.) in anhydrous DCM (1.0 M, 18.2 mL) pumped at 2.658 mL min^{-1}. The combined stream passed through two 10 mL reactor coils heated to 105 °C. System pressure was maintained using a 250 psi back pressure regulator.

Crude reaction mixture collected from the system was mixed vigorously with a saturated aqueous solution of ammonium chloride (30 mL). The organic phase was dried over magnesium sulfate and solvent removed under reduced pressure. DCM was slowly added until residues had dissolved completely, after which time the mixture was left until solvent had evaporated. Crystals of title compound (1.720 g, 8.7 mmol, 87 %) were collected as colourless needles.

Mp 148 - 149 °C (DCM) [Lit. 148 - 150 °C];[SI-2]

LC-MS t_R = 3.58 min, *m/z* 198.1;

IR (neat) ν/cm^{-1}: 3215 (bw), 3036 (w), 2973 (w), 1681 (w), 1643 (m), 1533 (m), 1476 (m), 1431 (m), 1376 (m), 1323 (m), 1249 (m), 1207 (m), 1147 (m), 980 (m), 795 (w), 760 (s), 708 (s), 663 (s);

^{1}H NMR (600 MHz, $CDCl_3$) δ/ppm = 7.86 (s, 1H, NH), 7.13 - 7.16 (m, 1H), 7.10 (d, J = 7.5 Hz, 2H), 4.24 (s, 2H), 2.24 (s, 6H);

^{13}C NMR (150 MHz, $CDCl_3$) δ/ppm = 164.30, 135.29, 132.64, 128.31, 127.82, 42.73, 18.24;

HRMS *m/z* calc. for $C_{10}H_{13}NOCl$ $[M + H]^+$ 198.0686, found 198.0688, Δ = 1.0 ppm;

Microanalysis calc. (found) for $C_{10}H_{13}NOCl$ C 60.77% (60.78%), N 7.09% (7.00%), H 6.12% (6.08%), Cl 17.93% (17.92%).

All recorded data were consistent with those reported previously.[SI-3]

2-(Diethylamino)-N-(2,6-dimethylphenyl)acetamide (8)

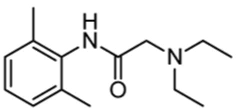

The equipment layout for this flow procedure is shown in the main text (Figure 3a).

A solution of compound 6 (0.988 g, 5.0 mmol) in DMF (0.5 M, 10 mL) was pumped at 0.570 mL min^{-1} to a tee junction where it mixed with a solution of diethylamine (2.02 mL, 19.5 mmol, 3.9 eq.) and TEA (2.72 mL, 19.5 mmol, 3.9 eq.) in DMF (2.0 m diethylamine, 5.01 mL) pumped at 0.556 mL min^{-1}. The mixture was directed through two 10 mL reactor coils heated to 99 °C. System pressure was maintained using a 100 psi back pressure regulator.

Crude reaction mixture was diluted with distilled water (25 mL) and DCM (25 mL) before it was agitated vigorously. The organic phase was washed with distilled water (3 x 10 mL) before volatiles were removed under reduced pressure. Residues were purified via flash column chromatography on silica (100 % ethyl acetate) to afford the title compound (1.148 g, 4.9 mmol, 98 %) as a light yellow solid.

Mp 67 - 68 °C (EtOAc) [Lit. 66 - 68 °C];[SI-4]

LC-MS t_R = 2.81 min, *m/z* 235.2;

IR (neat) *ν*/cm^{-1}: 3256 (bw), 2968 (m), 2923 (w), 2800 (w), 1737 (w), 1663 (s), 1594 (w), 1492 (s), 1423 (m), 1373 (m), 1292 (m), 1207 (m), 1165 (m), 1121 (m), 1091 (m), 1072 (m), 987 (m), 764 (s);

^{1}H NMR (600 MHz, $CDCl_3$) *δ*/ppm = 8.92 (s, 1H), 7.07 - 7.11 (m, 3H), 3.22 (s, 2H), 2.69 (q, J = 7.2 Hz, 4H), 2.23 (s, 6H), 1.14 (t, J = 7.2 Hz, 6H);

^{13}C NMR (150 MHz, $CDCl_3$) *δ*/ppm = 170.21, 135.03, 133.94, 128.17, 127.00, 57.50, 48.92, 18.53, 12.63;

HRMS *m/z* calc. for $C_{14}H_{23}N_2O$ [M + H]$^+$ 235.1810, found 235.1814, Δ = 1.7 ppm;

Microanalysis calc. (found) for $C_{14}H_{23}N_2O$ C 71.76% (71.56%), N 11.95% (11.76%), H 9.46% (9.59%).

All recorded data were consistent with those reported previously.[SI-4]

2-Bromo-1-(3-chlorophenyl)propan-1-one (10)

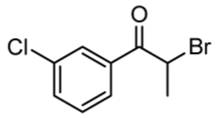

The equipment layout for this flow procedure is shown in the main text (Figure 4a).

A solution of 1-(3-chlorophenyl)propan-1-one (0.843 g, 5.0 mmol) in DCM (0.5 M, 10 mL) was pumped at 0.500 mL min^{-1} into a tee junction where it mixed with a solution of bromine (0.256 mL, 5.0 mmol, 1.0 eq.) in DCM (0.5 M, 10 mL) pumped at 0.500 mL min^{-1}. The combined solutions were directed through two 10 mL reactor coils heated to 44 °C. System pressure was maintained using a 75 psi back pressure regulator.

Collected reaction mixture was quenched with a saturated solution of sodium metabisulfite (30 mL), and the organic layer dried over magnesium sulfate. Removal of solvent afforded the title compound (1.238 g, 5.0 mmol, >99 %) as a colourless oil.

LC-MS t_R = 4.54 min, *m/z* 155.3;

IR (neat) *ν*/cm^{-1}: 1687 (s), 1570 (m), 1473 (w), 1442 (m), 1422 (m), 1376 (w), 1333 (m), 1230 (s), 1160 (m), 1076 (m), 996 (m), 959 (m), 901 (m), 801 (m), 786 (m), 738 (s), 696 (s), 672 (s);

^{1}H NMR (600 MHz, $CDCl_3$) *δ*/ppm = 7.99 (s, 1H), 7.89 (d, J = 7.9 Hz, 1H), 7.56 (d, J = 7.9 Hz, 1H), 7.43 (t, J = 7.9 Hz, 1H), 5.22 (q, J = 6.7 Hz, 1H), 1.90 (d, J = 6.7 Hz, 3H);

^{13}C NMR (150 MHz, $CDCl_3$) *δ*/ppm = 192.03, 135.62, 135.08, 133.55, 130.00, 128.96, 126.93, 41.24, 19.95;

HRMS *m/z* calc. for C_9H_9BrClO $[M + H]^+$ 246.9525, found 246.9524, Δ = -0.4 ppm;

Microanalysis calc. (found) for C_9H_9BrClO C 43.67% (43.67%), H 3.23% (3.10%).

All recorded data were consistent with those reported previously.[SI-5]

2-(tert-Butylamino)-1-(3-chlorophenyl)propan-1-one (12)

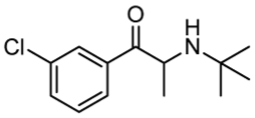

Single flow reaction

The equipment layout for this flow procedure is shown in the main text (Figure 5a).

A solution of compound 10 (0.619 g, 2.5 mmol) in N-methyl-2-pyrrolidone (NMP) (0.25 M, 10 mL) was pumped at 0.25 mL min^{-1} to a tee junction where it was mixed with a solution of *tert*-butylamine (0.788 mL, 7.5 mmol, 3.0 eq.) in NMP (0.25 M, 29.2 mL) pumped at 0.75 mL min^{-1}. The resulting solution was heated to 90 °C in two 10 mL reactor coils. System pressure was maintained using a 100 psi back pressure regulator.

Water (25 mL) was added to crude reaction mixture and the resulting solution extracted with diethyl ether (3 x 25 mL). The combined organic phases were washed with water (5 x 25 mL) before being dried over magnesium sulfate. Removal of solvent under reduced pressure following purification over silica (DCM/MeOH gradient elution, 0 % for 1 CV, 0 - 10 % over 5 CV, 10 % for 25 CV) afforded the title compound (0.479 g, 2.0 mmol, 80 %) as a yellow oil that solidified in the refrigerator.

Telescoped flow procedure

The equipment layout for this flow procedure is shown in the main text (Figure 6a). The parameters described herein are for steady state operation.

A solution of 1-(3-chlorophenyl)propan-1-one in DCM (0.5 M) was pumped at 0.5 mL min^{-1} to a tee junction where it mixed with a solution of bromine in DCM (0.5 M) pumped at 0.5 mL min^{-1} via peristaltic pump. The combined solution was directed through a 10 mL reactor coil held at 44 °C. System pressure was maintained using a 100 psi back pressure regulator.

Eluting crude reaction mixture was mixed with 2.0 mL min^{-1} (HPLC pump) of sodium metabisulfite solution in water (2.0 M) and passed through an Omnifit column (100 mm length, ⌀ 10 mm) filled with eight magnetic stirrer bars which was placed on top of a stirrer plate. Phases were separated in a vertical Omnifit column (100 mm length, ⌀ 10 mm) from which the organic layer was removed at an average rate of 0.500 mL min^{-1} via peristaltic pump and mixed with a stream of NMP at 0.500 mL min^{-1} (HPLC pump) at a tee junction.

The combined solution was passed into a distillation column held at 110 °C from which solution was drawn at 0.610 mL min^{-1} via peristaltic pump and mixed with 0.390 mL min^{-1} of *tert*-butylamine solution in NMP (1.928 M). The resulting mixture was directed through two 10 mL reactor coils heated to 90 °C. System pressure was maintained using a 100 psi back pressure regulator.

Solvent was removed from a 10 mL sample of crude reaction mixture under reduced pressure and residues were purified using the process described above for the single flow reaction to afford the title compound (0.479 g, 2.0 mmol, 80 %) as a yellow oil that solidified in the refrigerator.

LC-MS t_R = 3.43 min, *m/z* 240.2;

IR (neat) ν/cm^{-1}: 2967 (m), 1684 (s), 1571 (m), 1454 (m), 1424 (m), 1365 (m), 1298 (m), 1206 (s), 1142 (m), 1078 (m), 991 (m), 974 (m), 902 (w), 800 (m), 766 (m), 730 (s), 695 (m), 674 (s);

^{1}H NMR (600 MHz, $CDCl_3$) δ/ppm = 7.96 (s, 1H), 7.87 (d, J = 7.9 Hz, 1H), 7.55 (d, J = 7.9 Hz, 1H), 7.44 (t, J = 7.9 Hz, 1H), 4.29 (q, J = 7.2 Hz, 1H), 2.31 (s, 1H), 1.25 (d, J = 7.2 Hz, 3H), 1.04 (s, 9H);

^{13}C NMR (150 MHz, $CDCl_3$) δ/ppm = 203.80, 136.64, 135.15, 133.18, 130.09, 128.42, 126.36, 52.22, 50.80, 29.73, 22.48;

HRMS *m/z* calc. for $C_{13}H_{19}NClO$ $[M + H]^+$240.1150, found 240.1142, Δ = -3.3 ppm;

Microanalysis calc. (found) for $C_{13}H_{19}NClO$ C 56.53% (56.55%), N 5.07% (5.07%), H 6.93% (6.95%), Cl 25.67% (25.42%). Note: elemental analysis of the hydrochloric salt of compound 12 was performed owing to instability of the free base.

All recorded data were consistent with those reported previously.[SI-6, SI-7, SI-8]

(1S,2S)-2-((dimethylamino)methyl)-1-(3-methoxyphenyl)cyclohexan-1-ol (3) NMR

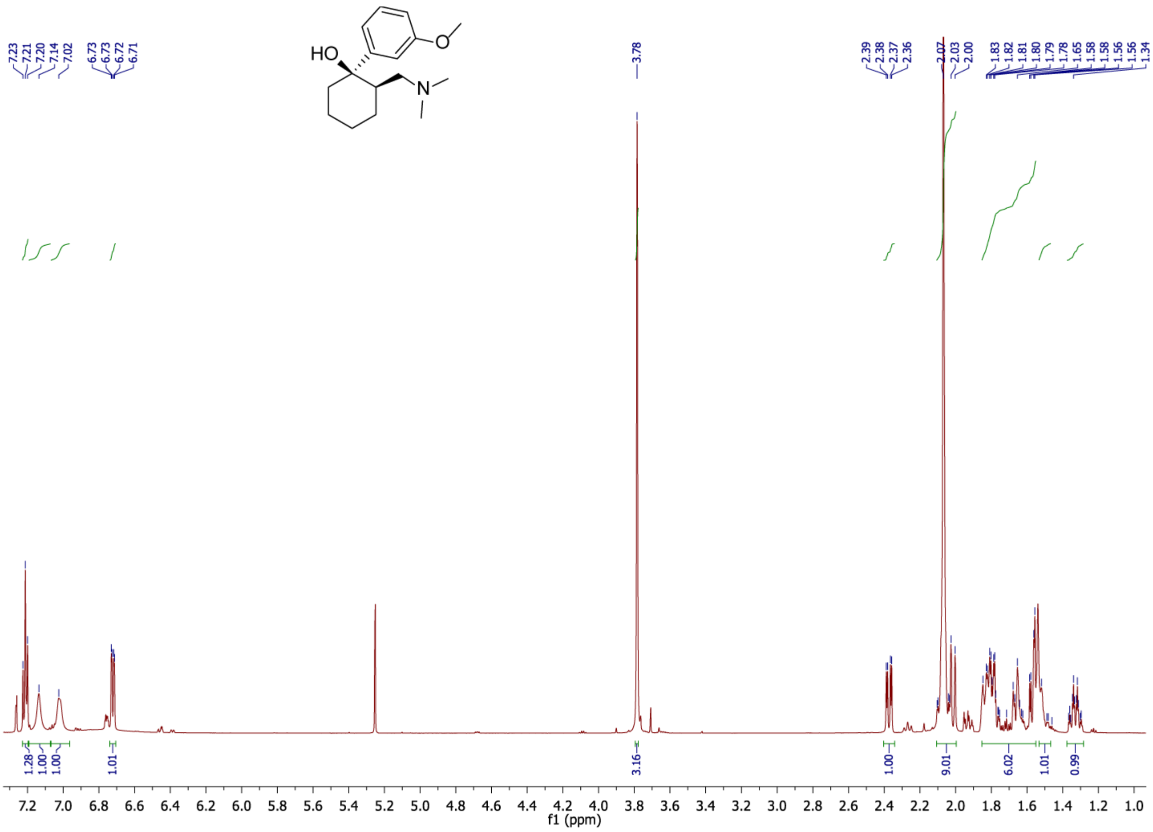

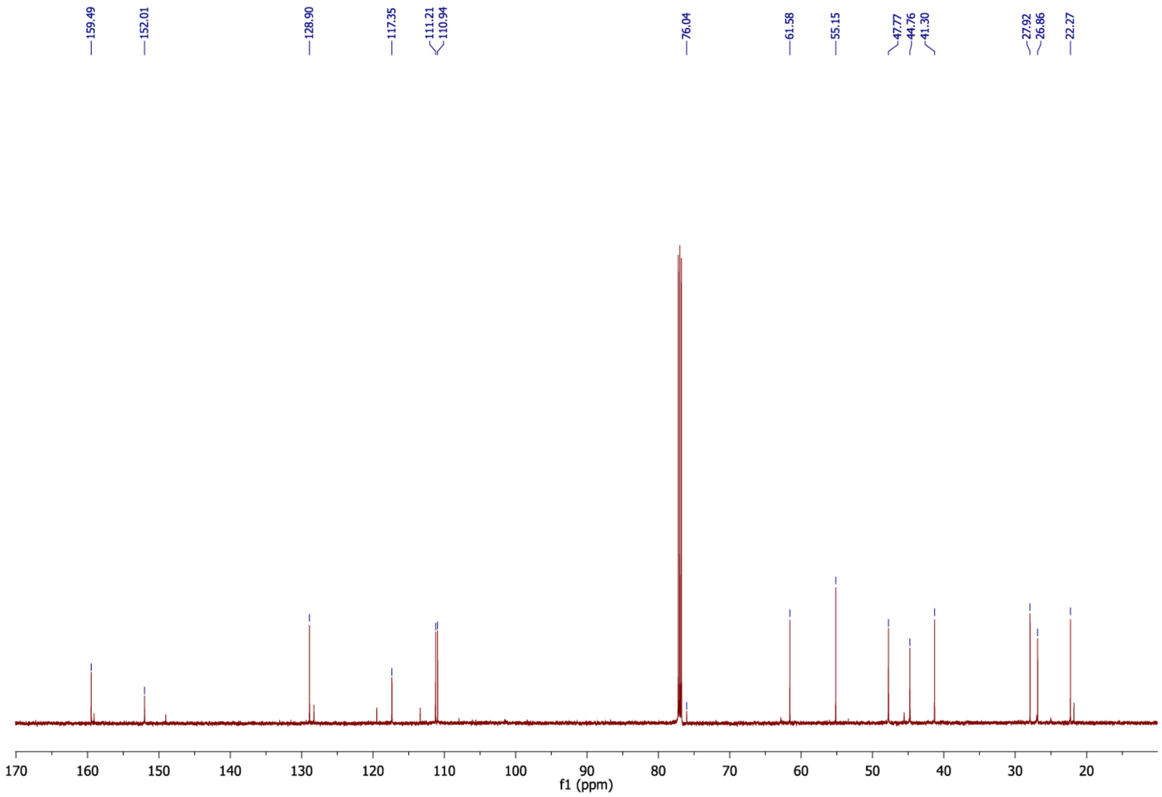

2-Chloro-N-(2,6-dimethylphenyl)acetamide (6) NMR

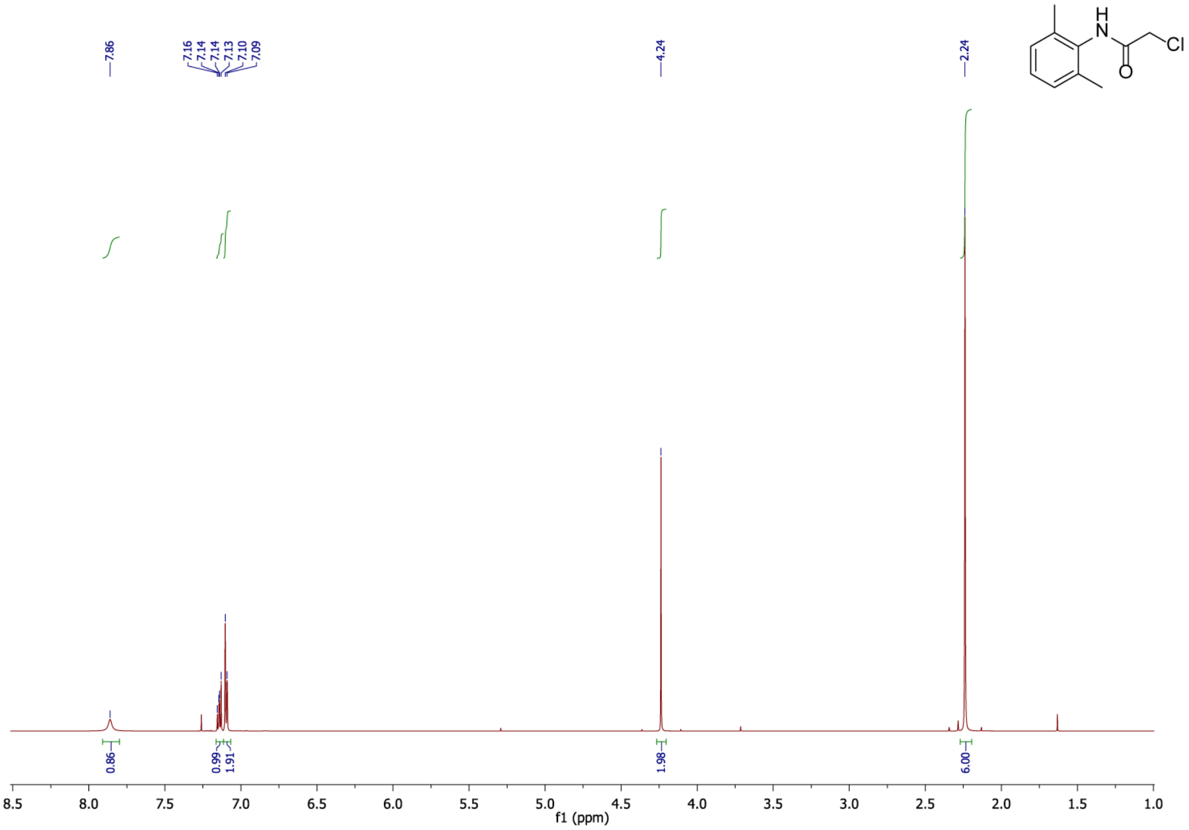

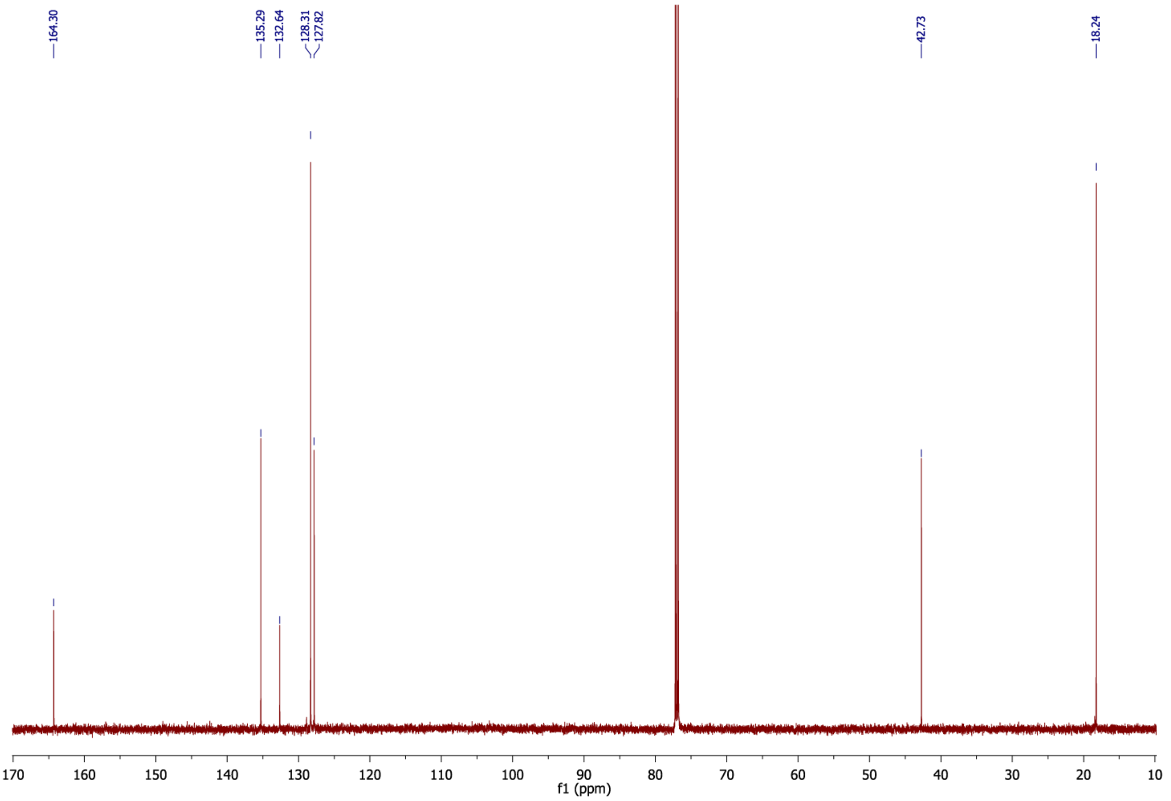

2-(Diethylamino)-N-(2,6-dimethylphenyl)acetamide (8) NMR

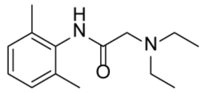

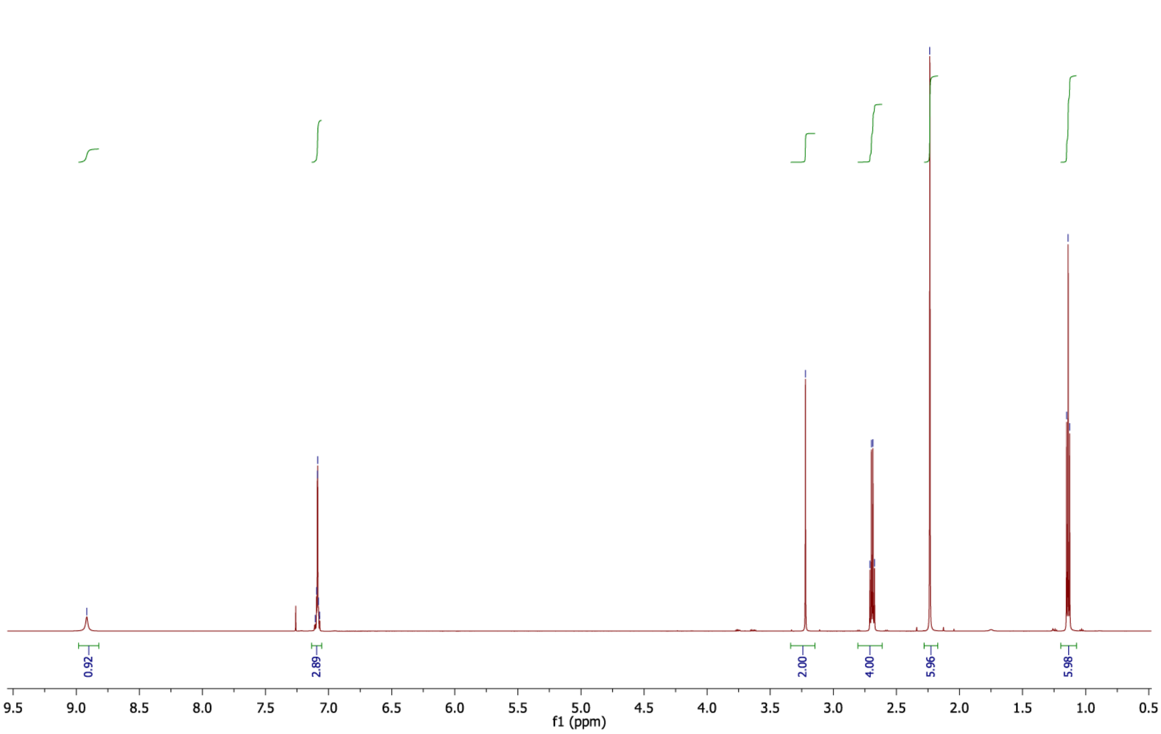

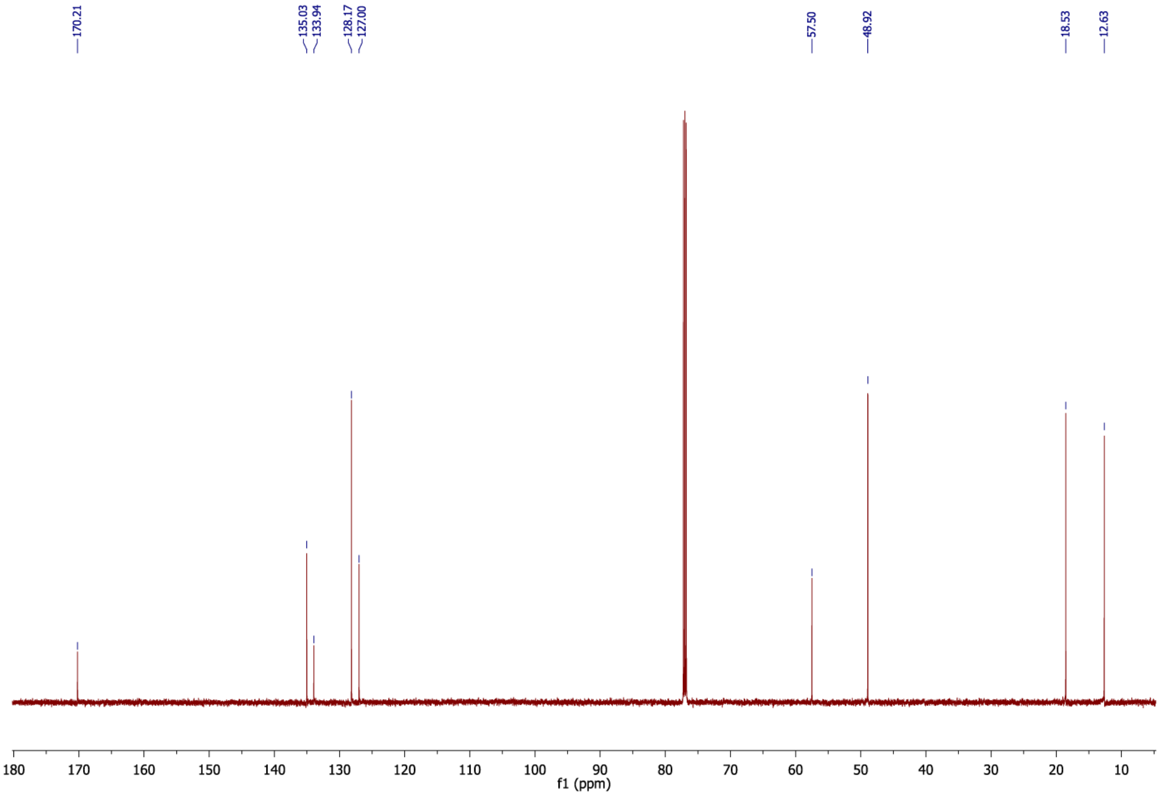

2-Bromo-1-(3-chlorophenyl)propan-1-one (10) NMR

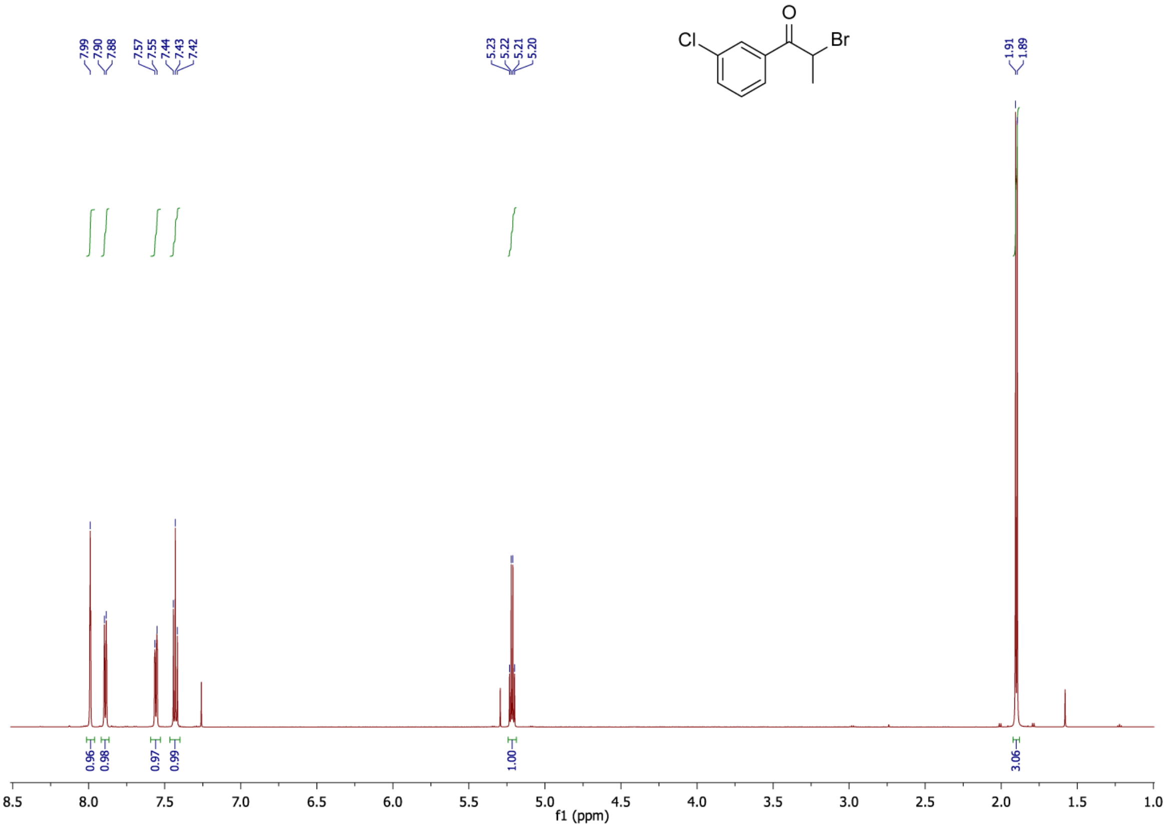

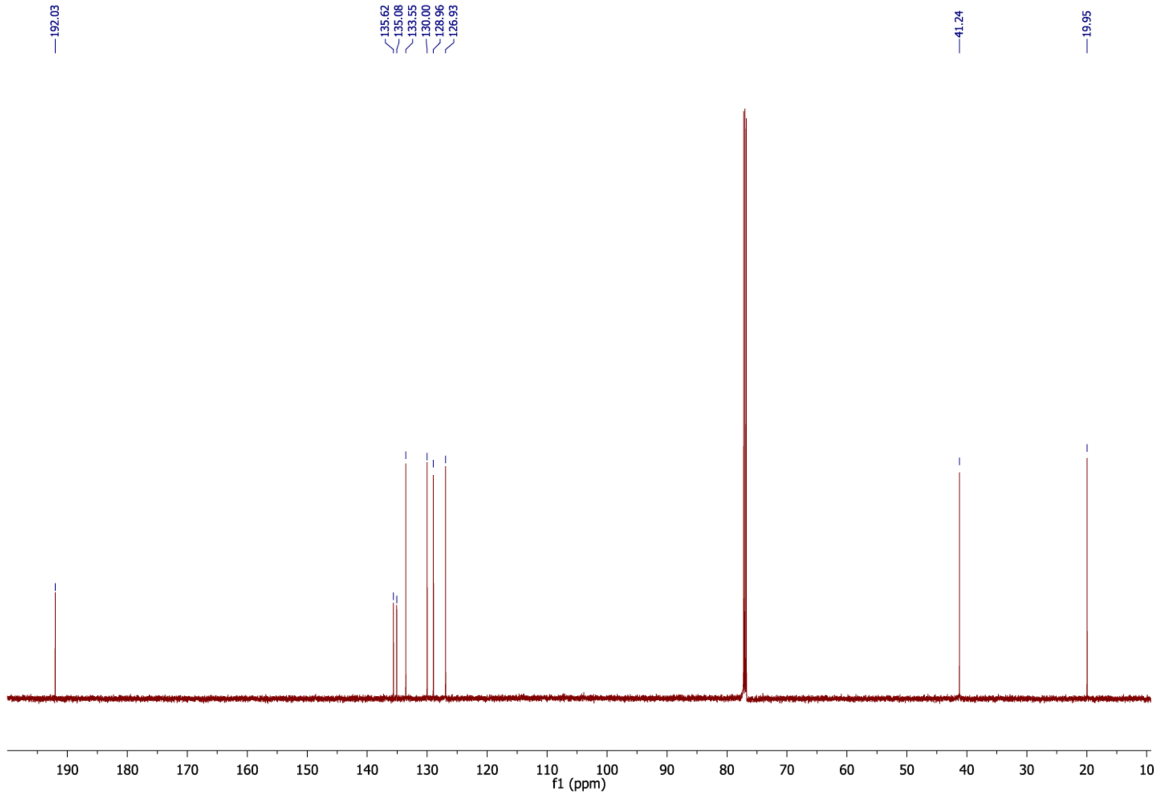

2-(tert-Butylamino)-1-(3-chlorophenyl)propan-1-one (12) NMR

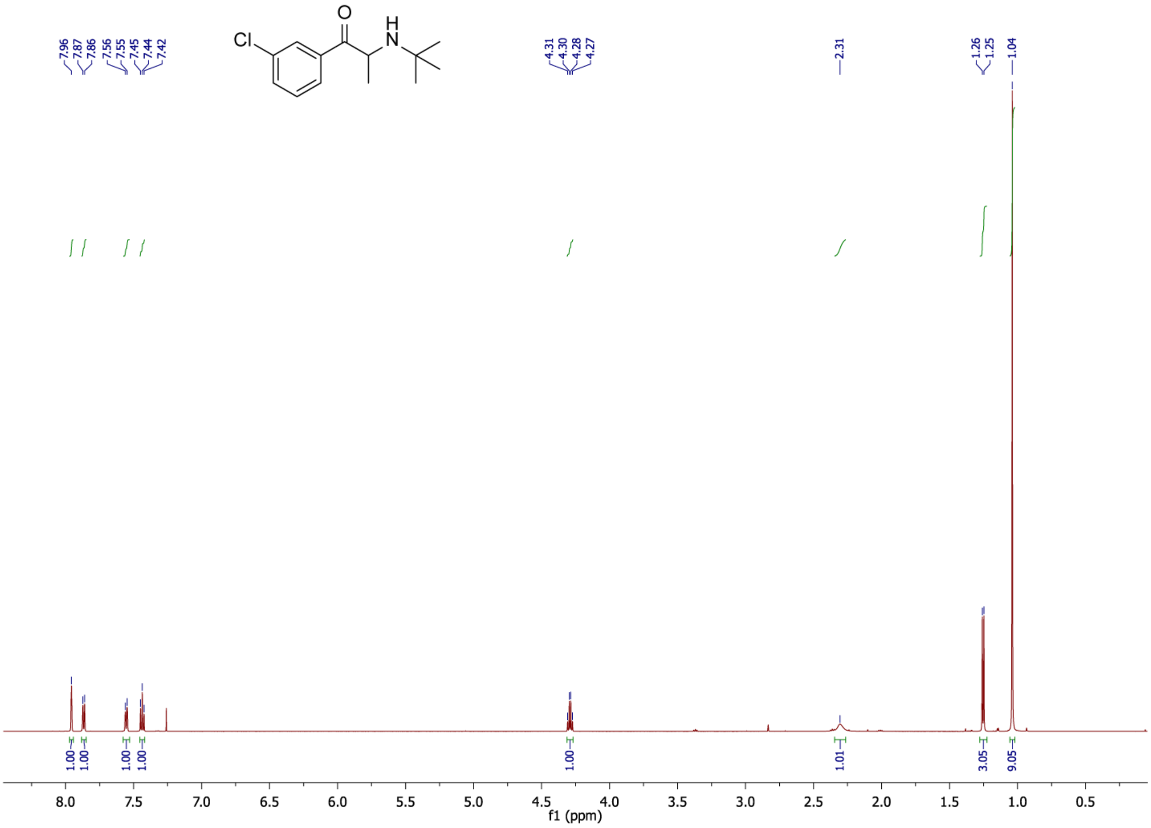

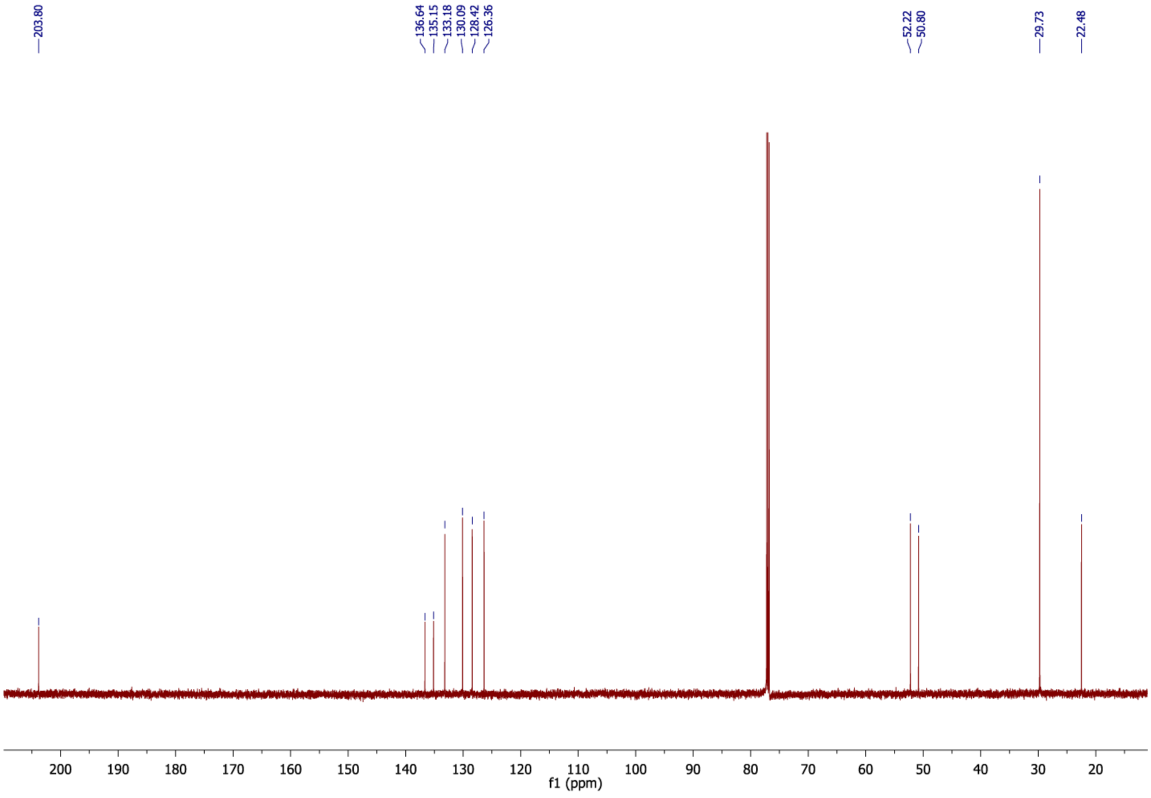

5.0 References

[SI-1] D.E. Fitzpatrick and S.V. Ley, *React. Chem. Eng.* **2016**, *1*, 629-635.

[SI-2] J. Lázár and G. Bernáth, *J. Heterocycl. Chem.* **1990**, *27*, 1885-1892.

[SI-3] R.J. Rahaim and R.E. Maleczka, *Synthesis*, **2006**, *2006*, 3316-3340.

[SI-4] L. Zhao, L. Fang, Y. Xu, S. Liu, Z. He and Y. Zhao, *Eur. J. Pharm. Biopharm.* **2008**, *69*, 199-213.

[SI-5] A. K. Ghorpade, S. N. Huddar and K. G. Akamanchi, *Tetrahedron Lett.* **2016**, *57*, 4918-4921.

[SI-6] Data were compared with those held by the National Institute of Advanced Industrial Science and Technology (AIST), Japan. SDBD record number 52948.

[SI-7] M. O. Hamad, P. K. Kiptoo, A. L. Stinchcomb and P. A. Crooks, *Bioorg. Med. Chem.* **2006**, *14*, 7051-7061.

[SI-8] G. W. Amarante, M. Cavallaro and F. Coelho, *J. Braz. Chem. Soc.* **2011**, *22*, 1568-1584.
